# The Applicability of Chromatographic Retention Modeling on Chiral Stationary Phases in Reverse-Phase Mode: A Case Study for Ezetimibe and Its Impurities

**DOI:** 10.3390/ijms242216097

**Published:** 2023-11-08

**Authors:** Elek Ferencz, Éva-Katalin Kelemen, Mona Obreja, Gergő Tóth, Melinda Urkon, Arnold Zöldhegyi, Emese Sipos, Zoltán-István Szabó

**Affiliations:** 1 Department of Physical Chemistry, Faculty of Pharmacy, George Emil Palade University of Medicine, Pharmacy, Science, and Technology of Targu Mures, 540142 Targu Mures, Romania; elek.ferencz@umfst.ro; 2 Gedeon Richter Romania S.A., Analytical Development Department, 540306 Targu Mures, Romania; 3 Department of Pharmaceutical Chemistry, Semmelweis University, 1083 Budapest, Hungary; 4 Department of Pharmacology and Clinical Pharmacy, George Emil Palade University of Medicine, Pharmacy, Science, and Technology of Targu Mures, 540142 Targu Mures, Romania; 5 Molnár-Institute for Applied Chromatography, 10407 Berlin, Germany; 6 Department of Pharmaceutical Industry and Management, George Emil Palade University of Medicine, Pharmacy, Science, and Technology of Targu Mures, 540142 Targu Mures, Romania; 7 Sz-imfidum Ltd., 525401 Lunga, Romania

**Keywords:** AQbD, DryLab, chiral stationary phase, experimental design, retention modeling, mechanistic model, related substances, ezetimibe

## Abstract

Mechanistic modeling is useful for predicting and modulating selectivity even in early chromatographic method development. This approach is also in accordance with current analytical quality using design principles and is highly welcomed by the authorities. The aim of this study was to investigate the separation behavior of two different types of chiral stationary phases (CSPs) for the separation of ezetimibe and its related substances using the mechanistic retention modeling approach offered by the Drylab software (version 4.5) package. Based on the obtained results, both CSPs presented with chemoselectivity towards the impurities of ezetimibe. The cyclodextrin-based CSP displayed a higher separation capacity and was able to separate seven related substances from the active pharmaceutical ingredient, while the cellulose-based column enabled the baseline resolution of six impurities from ezetimibe. Generally, the accuracy of predicted retention times was lower for the polysaccharide CSP, which could indicate the presence of additional secondary interactions between the analytes and the CSP. It was also demonstrated that the combination of mechanistic modeling and an experimental design approach can be applied to method development on CSPs in reverse-phase mode. The applicability of the methods was tested on spiked artificial placebo samples, while intraday and long-term (2 years) method repeatability was also challenged through comparing the obtained retention times and resolution values. The results indicated the excellent robustness of the selected setpoints. Overall, our findings indicate that the chiral columns could offer orthogonal selectivity to traditional reverse-phase columns for the separation of structurally similar compounds.

## 1. Introduction

Retention modeling is a science-based approach to chromatographic method development. Generally, it is based on a systematic experimental framework which allows a deeper understanding of the separation process by revealing the correlations between analytical procedure parameters and quality attributes. There are numerous computer-assisted tools for data processing, such as empirical-model-based software for experimental design (DoE—design of experiments) and statistical analysis (for ex. Design Expert or Modde) [1,2,3,4], or theoretical-model-based software packages using chromatographic first-principles such as the linear solvent strength model (LSSM) or the solvophobic theory for the prediction of the chromatographic behavior of examined substances (for ex. DryLab, Molnar Institute, Berlin, Germany) [5,6,7,8,9]. One characteristic that these two different approaches have in common is that both use systematic preliminary experiments as input data for the construction of the virtual separation models. These procedures are in line with the recently published point of view of the regulatory authorities in the draft versions of the ICH Q2(R2) [10] and Q14 [11] guidelines regarding Analytical Quality by Design (AQbD) principles. The ICH Q14 guidance presents the “enhanced approach” of the method development process, which includes the identification of analytical procedure parameters (APPs) that can impact method performance, the realization of uni- or multi-variate experiments to explore ranges and interactions between APPs, and the establishment of analytical procedure control strategy based on enhanced process understanding. A definition of a lifecycle management plan, including proven acceptable ranges (PARs), established conditions (ECs), and method operational design regions (MODRs), is also presented.

The main advantage of employing an “enhanced approach” through software-assisted method development is acquiring an understanding of the process through the identification of critical method parameters (CMPs or ECs) and critical quality attributes (CQAs), which are essential for establishing a control strategy. There are numerous examples from recent years about the applicability of experimental design-based retention modeling in chromatographic method development and optimization using both empirical-model-based, statistical- [2,4,12,13,14,15,16,17,18,19,20,21] and theoretical-model-based, or mechanistic [8,22,23,24,25,26] approaches.

The DryLab software package relies on fundamental chromatographic theories, such as the solvophobic theory described by Csaba Horváth and Imre Molnár [27,28] and LSSM [29], alongside the experimental design-based framework for chromatographic retention predictions. The software is widely applied mostly for modeling reverse-phase separation processes [24,30,31,32,33,34], but is also useful for the optimization of ion-exchange chromatography (IEX) [35], hydrophobic interaction chromatography (HIC), and hydrophobic interaction liquid chromatography (HILIC) [8,36,37]. Consequently, the utilization of this widely recognized theoretical model in the software enables accurate anticipation of the chromatographic performance of analytes in reverse-phase mode. The software uses preliminary experiments to fine-tune the theoretical model, raising the accuracy of the prediction. Nevertheless, in chiral chromatography, such circumstances are infrequent, and the predictability of the outcome of chiral separations is often limited, because of the superposition of the chemo- and stereoselective effects during the separation process. This is further complicated by the changes in the solvation and three-dimensional structure of some of the chiral selectors.

In the last few years, several studies have underlined the utility of chiral stationary phases (CSPs) in providing orthogonal selectivity to traditional reverse-phase columns. In the case of ivabradine, both chiral and achiral impurities were simultaneously separated on a cellulose-based column using the polar organic mode [4]. Tome T. et al. published a study in 2020 about the separation of process-related impurities of celecoxib, where various C18- and phenyl-type reverse-phase columns were screened, but the proper selectivity was achieved only using an amylose-based column in reverse-phase mode [2]. In 2023, the simultaneous separation of achiral and chiral impurities of vildagliptin was achieved by Papp L. et al. using a cellulose-based chiral selector in reverse-phase mode [38]. Recently, another study was published about method development for the determination of chiral impurity of rivaroxaban in reverse-phase mode, using the statistical approach for optimization as a tool of the quality by design concept [39]. The simultaneous separation of achiral and chiral escitalopram impurities was realized on a cellulose-based CSP in reverse-phase mode [40]. All the above-mentioned methods were developed using empirical-model-based approaches for method optimization, demonstrating the applicability of statistical modeling on CSPs. However, the United States Pharmacopeia (USP) 1220 general monograph states, “When available, mechanistic models can be used to understand the effect of procedure parameters on performance. Use of mechanistic models can reduce experimental work and provide a reliable estimate of the behavior of the analytes of interest” [41]. The statistical approach is generally recommended in cases where the major interactions between the method parameters and quality attributes are unknown, but this is not the case for reverse-phase separation processes. Therefore, in this case, the mechanistical modeling probably delivers more accurate multivariate separation models, in comparison with the statistical approach.

The aim of this study was to investigate the applicability of mechanistic modeling based on an experimental design framework for the separation of achiral-related substances of ezetimibe on different types of CSPs, with a special emphasis on the predictability of the theoretical separation models constructed by the DryLab software (version 4.5). Ezetimibe is a serum cholesterol-lowering agent, indicated for the treatment of primary hypercholesterolemia or mixed dyslipidemia. Through its interaction with the NPC1L1 transporter, it inhibits intestinal cholesterol (dietary and biliary) and phytosterol absorption. This complementary mechanism of action enhances the efficacy of HMG-CoA reductase inhibitors; however, it is also used in monotherapy [42]. Because of the complex anazetidinone structure of the drug, several different synthetic pathways are possible [43,44]; consequently, numerous related substances are available, such as synthesis intermediates or degradation products of the active substance. In our previous study, a simple chromatographic method was developed, which can be used in routine quality control for the determination of the most relevant process-related impurities and degradation products of ezetimibe [9], but in this case, the main goal was to demonstrate the applicability and accuracy of mechanistic modeling in the case of CSPs; therefore, two other more special process-related impurities (monofluoro ezetimibe and RRS ezetimibe) were included in the study. The proper separation of ezetimibe and its related substances is challenging because of the highly similar chemical structure of the analytes including very similar chemical structures from low to high hydrophobicity, while the use of CSPs might provide the necessary orthogonal selectivity compared to “traditional” reverse-phase columns.

## 2. Results and Discussion

### 2.1. Results of the Preliminary Experiments

It was observed and demonstrated by previous studies that, in the case of chiral columns, the recognition pattern of the stationary phases is significantly influenced by temperature [45,46,47,48,49,50]. As a result, during the initial experimental runs, lower and higher column temperatures were tested. The separation performance (resolution and the number of separated impurities) was better around 30 °C compared to higher temperatures; therefore, the high temperature of the design framework was set at T_2_ = 35 °C and T_1_ = 5 °C was selected as the lowest temperature, to maintain the required temperature difference of corner runs as suggested by the software. Generally, in the case of enantioseparation, the temperature has a non-linear effect on the selectivity and the elution order because of the temperature-induced structural transitions of the chiral stationary phases [45].

Regarding the gradient time, several values were tested between 10 and 80 min. Obviously, the higher the gradient time the better the separation performance, but it is necessary to consider that extremely high analysis times are inadequate for routine use. Based on these aspects, 20 min was selected as the short gradient time (tG_1_ = 20 min); considering the three-fold difference, the long gradient time was set as 60 min (tG_2_ = 60 min). Because of the high retention of the analytes, the gradients were started with 30% organic mobile phase, rising continuously to 100% organic solvent.

Generally, in the case of chiral chromatography, the elution order of the examined substances is highly influenced by the composition and type of the organic solvent used [51,52,53,54,55,56,57,58]. This is the reason why the full range from 100% ACN to 100% MeOH was modeled through the DoE, instead of the 100% ACN to 60% MeOH used in our previous study for comparison of reverse-phase stationary phases selectivity in the case of ezetimibe and its related substances [9]. Modification of the ternary composition of the organic mobile phase during the preliminary runs shows that neat ACN and neat MeOH offer different separation patterns; therefore, the full range from 100% MeOH to 100% ACN was covered with three different tC levels (tC_1_ = 100% ACN, tC_2_ = 50% MeOH in ACN and tC_3_ = 100% MeOH) during the experimental design.

After the preliminary phase, the selected experimental design with the established CMP intervals was realized on both stationary phases.

### 2.2. The Separation Model for the Chiralcel OD Column

The chromatograms obtained from the different conditions of the experimental design were used as input data for the DryLab software (version 4.5) to build up the virtual separation model. Table S1 summarizes the obtained retention times in the 12 corner conditions for all substances.

**Figure 1:**
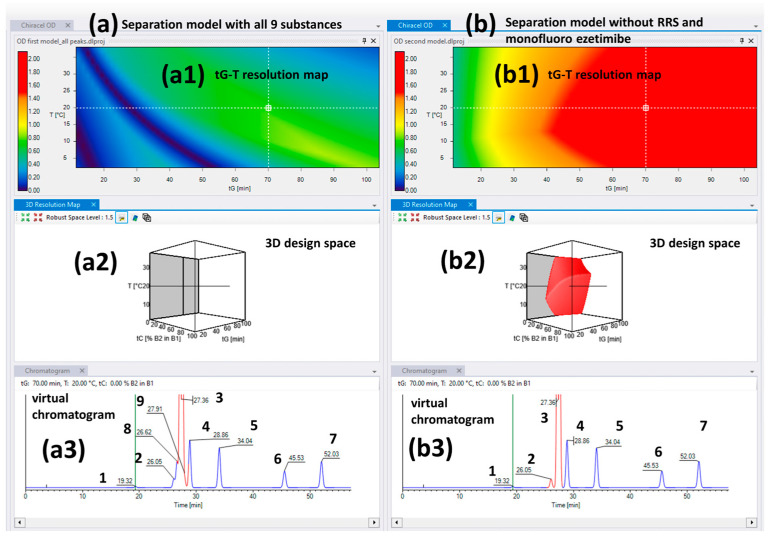
The virtual separation models for the Chiralcel OD column (**a2**,**b2**) and the 2D tG-T resolution maps (**a1**,**b1**) in the case of 100% ACN as organic mobile phase; (**a**) first model including all nine peaks but without design space with baseline separation between desfluoro ezetimibe, RRS ezetimibe, ezetimibe API, and monofluoro ezetimibe, respectively, (**b**) second model withoutRRS and monofluoro ezetimibe impurities, but the baseline separation of the remaining seven peaks is ensured. The virtual chromatograms obtained in the same conditions from both models are represented as (**a3**,**b3**) (**1**—ezetimibe diol; **2**—desfluoro ezetimibe; **3**—ezetimibe API; **4**—THP compound; **5**—ezetimibe ketone; **6**—TBDMS ketone; **7**—benzylated ezetimibe; **8**—RRS ezetimibe; **9**—monofluoro ezetimibe).

Based on the obtained retention times represented in Table S1 several selectivity problems can be identified. Firstly, RRS-ezetimibe elutes very close to ezetimibe in all 12 tested conditions, and in some cases, it elutes close to or even co-elutes with desfluoro ezetimibe. Another selectivity issue is between the monofluoro ezetimibe and the THP compound which have similar retention times in all conditions. The obtained separation models are visible in Figure 1a including all nine peaks and (b) without the RRS and monofluoro ezetimibe impurities. The 3D design spaces represented on Figure 1(a2,b2) is practically a heat-map type of visual representation of the critical resolution (R_s,crit._—resolution between the closest eluted peaks) as a function of the modeled method parameters (tC, tG, and T). As observed in case of Figure 1(a2), the whole 3D area is empty, without red zones which means that there are no method parameter combinations in the studied intervals that can ensure the baseline separation (R_s,crit._ ≥ 1.5) of all peaks.

It becomes clear that the Chiralcel OD column is not able to separate all the desired analytes; therefore, in turn, we excluded the problematic peaks to obtain a functional separation model, with a useful design space, where the remaining peaks are properly separated. Therefore, we excluded RRS ezetimibe because of its close retention time to ezetimibe. Another problematic peak pair was that of monofluoro ezetimibe and THP compound. As the next step, monofluoro ezetimibe was excluded, as a process-related impurity, instead of the THP compound, which is the most important degradation product, and it has higher practical importance in comparison with the monofluoro compound. The second virtual separation model obtained after the exclusion of the above-mentioned two impurities is represented in Figure 1b. According to this separation model, six related substances were baseline separated in the presence of ezetimibe on the Chiralcel OD column.

To demonstrate the validity of this model, different conditions were selected from the MODR, visualized in Figure 1(b2), and these setpoints were also run experimentally. The obtained results (retention times and resolutions) were compared with the software’s predicted values. The obtained results are summarized in Table 1.

The first two setpoints are similar in the slope of the gradient. The first one is a longer gradient at a lower temperature; therefore, the analysis time is also longer. Although the experimentally obtained R_s,crit._ is very close to the predicted value, there are differences between the experimentally obtained and model-predicted retention times, with an average retention time error of 5.88%. The second setpoint is characterized by the steepest gradient. Using this setpoint, the baseline separation of all peaks is not achieved; however, the correlation between the predicted vs. experimentally obtained retention times and enantioresolution is the closest (average retention time error is 1.27%).

The third setpoint was a gradient with two steps to verify the prediction power of the model using a more complicated gradient program. Theoretically, based on the DryLab calculation this setpoint is not able to baseline separate desfluoro ezetimibe and the API, which was also confirmed with the experimental run (R_s,crit._ < 1.5). For the selection of the last setpoint, a new DS model was calculated by including the monofluoro ezetimibe and excluding the THP compound. Based on this new model without the THP compound, the monofluoro ezetimibe can be separated from the API using 100% MeOH as the organic part of the mobile phase. This was demonstrated by the experimental run of setpoint 4.

It is also important to note that the corner runs were realized with a 0.5 mL/min flowrate and all the tested setpoints were conducted with 0.7 mL/min to speed up the analysis, further demonstrating the prediction power of the separation model. In all four cases, good correlations were observed between the experimental and predicted retention times, meaning that this type of flowrate extrapolation is working well. The correlation between the theoretical and experimental results (retention times and critical R_s,crit._ values) was good, and only slight differences in retention times were observed, demonstrating the validity of the virtual separation model.

**Figure 2:**
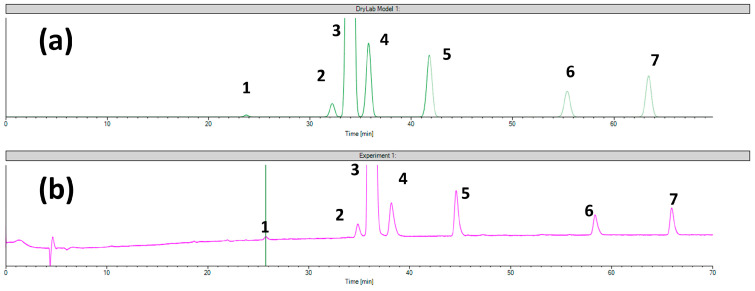
The comparison of (**a**) virtual chromatogram generated by the DryLab and (**b**) the experimental chromatogram obtained in case of setpoint 1 tested for Chiralcel OD column (**1**—ezetimibe diol; **2**—desfluoro ezetimibe; **3**—ezetimibe API; **4**—THP compound; **5**—ezetimibe ketone; **6**—TBDMS ketone and **7**—benzylated ezetimibe).

Setpoint 1 ensures the highest critical resolution with the highest number of separated peaks (six impurities and the API), but it took 70 min. This analysis time is not feasible for routine application. Figure 2 represents the comparison of the virtual chromatogram generated by the software and the experimentally obtained one. As can be observed in the experimentally obtained chromatograms, all peaks elute later than the software predicts; there is a noticeable retention time shift. However, it is also noteworthy that an excellent correlation can be observed between the predicted and experimentally obtained selectivity and resolution values. This means that the software can still be used for complex sample analysis on polysaccharide CSP in reverse-phase mode for finding setpoints, where baseline resolution can be achieved, with the possible limitation that retention time prediction would not be accurate in all cases.

Differences between the predicted and experimentally obtained retention times on different CSPs were described earlier by several research groups. Lämmerhofer et al. described the Drylab-based optimization of the simultaneous enantioseparation of seven derivatized amino acids on a quinine carbamate-type CSP [58]. Several linear- and multi-segmented, organic modifier and buffer salt gradients were software-simulated and experimentally verified. The average error of prediction for retention times varied between 2.69 and 8.02%. The higher retention time errors were explained that apart from reverse-phase elution, ion-exchange, and enantiospecific retention mechanisms are also present in these separation systems. Nevertheless, the authors concluded that the software was helpful in the optimization of the simultaneous enantioseparation of the seven racemic derivatized amino acids, reducing the analysis time from 230 min to 65 min [59,60].

Wagdy et al. studied the predictability of chiral separation of several racemic compounds on macrocyclic antibiotics-based columns (Chirobiotic V and T), polysaccharide CSP (Chiralpak AD-RH), and the protein-based Ultron ES-OVM. Drylab was able to predict the chromatographic retention of several compounds on the Chirobiotic V column, as it was characterized by a typical reverse-phase retention mechanism. However, on the other columns, due to the participation of other types of interactions on analyte retention, poor prediction capability was observed. The prediction was especially poor on the polysaccharide-type Chiralpak AD-RH column, which is based on amylose tris(3,5-dimethylphenylcarbamate) chiral selector. The authors hypothesized that the significance of the hydrogen–bond interactions hindered the ability of Drylab to accurately predict the retention behavior of the enantiomers [61].

In a similar study, the same group described the inability of Drylab to predict the retention behavior and chiral resolution of racemic amino acids on a ristocetin A-based CSP. The authors conclude that this was due to the hydrophilic interaction-based retention behavior of the analytes, rather than being due to the reverse-phase mechanism [62].

### 2.3. The Separation Model for the Chiral CD-Ph Column

Similarly to the Chiralcel OD column, a virtual separation model was realized for the Chiral CD-Ph column based on the 12 corner runs of the experimental design. To demonstrate the changes in the retention times in the function of the method parameters the experimentally obtained values (retention times and critical resolutions) are summarized in Table S2. In comparison with the Chiralcel OD column, the cyclodextrin-based CSP has a lower retentivity in all cases, which is beneficial from the point of view of the total analysis time.

**Figure 3:**
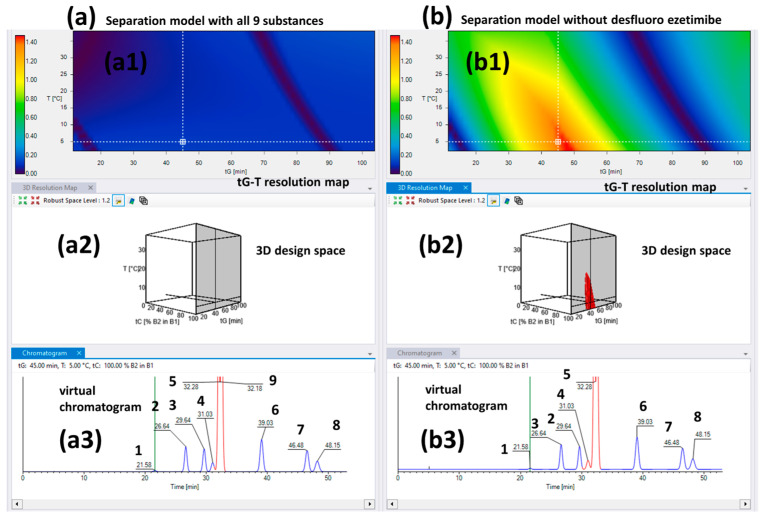
The virtual separation models for the Chiral CD-Ph column (**a2**,**b2**) and the 2D *tG-T* resolution maps (**a1**,**b1**) in the case of 100% MeOH as organic mobile phase; (**a**) includes all nine peaks but without design space with baseline separation between ezetimibe API and desfluoro ezetimibe, respectively; (**b**) is a model without desfluoro ezetimibe but the baseline separation of the remaining eight peaks is ensured. The virtual chromatograms obtained in the same conditions from both models are represented as (**a3**,**b3**) (**1**—ezetimibe diol; **2**—THP compound; **3**—RRS ezetimibe; **4**—monofluoro ezetimibe; **5**—ezetimibe API; **6**—ezetimibe ketone; **7**—TBDMS ketone; **8**—benzylated ezetimibe; **9**—desfluoro ezetimibe).

The obtained three-dimensional separation models are represented in Figure 3a,b. As in the case of the Chiralcel OD column, no setpoints were found for the baseline separation of all nine peaks (Figure 3(a1,a2)). Thus, further refinement was carried out by excluding desfluoro ezetimibe because it overlapped with the API in numerous corner runs (Figure 3(b1)). An optimal condition was selected from the first model and the virtual chromatogram was visualized, and it was shown that the desfluoro ezetimibe was co-eluted with the ezetimibe API. The virtual chromatogram for the same conditions was extracted from the second model too, where it can be viewed that the retention times are the same, only the status of the critical peak pairs (marked with red) is changed. This comparison was performed to demonstrate that the exclusion of the peaks has no effect on the retention model, but the second model is more useful for method optimization regarding the identification of MODR and control strategy establishment.

Validation of the virtual model was realized with four selected setpoints, which were run experimentally to compare the obtained data with the predicted ones. The conditions for the tested setpoints and the obtained results are detailed in Table 2.

In the case of the Chiral CD-Ph column, based on the virtual model, the high content of MeOH and a very low temperature were able to ensure better chromatographic performance. The first setpoint was selected to separate the highest possible number of impurities. Seven related substances were resolved from the API. The minimum critical R_s_ value observed was 1.33 between monofluoro ezetimibe and ezetimibe. The second setpoint had a lower number of related substances (without monofluoro ezetimibe compared to setpoint 1) but in exchange, higher robustness was obtained. The third setpoint was selected to demonstrate the precision of flow extrapolation; from the original 0.5 mL/min used in the corner experiments, 0.7 mL/min was tested. The last setpoint was chosen to verify the precision of retention time prediction in case of changes in the elution order. Based on the model using the condition of setpoint 4 the TBDMS ketone and benzylated ezetimibe are replaced regarding their elution order. This theory was confirmed by the experimental chromatogram. The correlation between the virtual and experimental data is excellent in all four cases, confirming the validity and prediction power of the 3D virtual separation model. The relationship between the experimental and predicted retention times for all four tested setpoints is represented in Figure S1.

The average of retention time errors for all peaks is 2.23% and in the case of the CD-Ph column this is 4.22%, in the case of Chiralcel OD CSP, meaning that the separation model is more accurate in the case of the CD-Ph column. As also discussed in Section 3.2, this could be due to additional, secondary interactions involved in the case of the cellulose-based chiral column, apart from the ones described in the solvophobic theory and LSSM. On the other hand, the retention mechanism on CD-based CSPs in reverse-phase mode, especially the mechanism of inclusion complexation, is more similar to the principles of solvophobic theory, which could be the reason for the higher correlation on this CSP. However, further experimental verification is needed to support this affirmation. Nonetheless, similarly good correlations between predicted and experimentally obtained chromatographic behavior of four atropoisomers on a Cyclobond I 2000 HP-RSP column, based on the *R,S*-hydroxypropyl-ether derivative of β-CD, which further underlines our observations [63].

**Figure 4:**
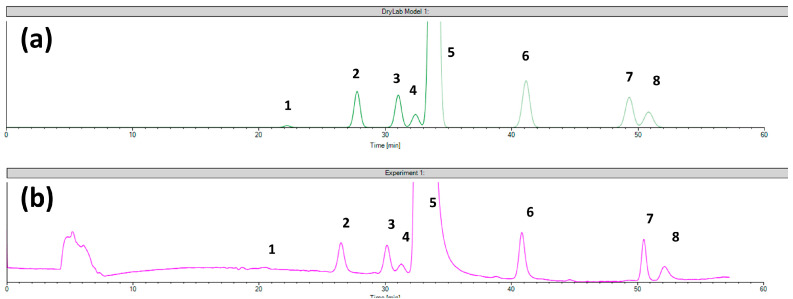
The comparison of (**a**) the virtual chromatogram generated by the DryLab and (**b**) the experimental chromatogram obtained in the case of setpoint 1 tested for Chiral CD-Ph column (**1**—ezetimibe diol; **2**—THP compound; **3**—RRS ezetimibe; **4**—monofluoro ezetimibe; **5**—ezetimibe API; **6**—ezetimibe ketone; **7**—TBDMS ketone; **8**—benzylated ezetimibe).

The virtual and experimental chromatograms of setpoint 1 are shown in Figure 4 demonstrating good retention time correlation but the pure selectivity without baseline separation between the RRS ezetimibe, monofluoro ezetimibe, and ezetimibe peaks. An advantage of the CD-Ph column was that in this case was realized the separation of monofluoro ezetimibe and RRS ezetimibe from other impurities using neat MeOH as an organic mobile-phase component, which was not possible using the Chiralcel OD CSP.

### 2.4. Applicability of the Methods and Practical Relevance

The applicability of the developed methods was tested in more realistic circumstances, using an artificial sample, which contained all the placebo components of the commercially available ezetimibe tablet formulation, along with ezetimibe at 1000 µg/mL concentration and the specific impurities in function of the selected setpoints. To investigate long-term repeatability of the method, the selected setpoints (see below) were repeated 2 years after the initial method development phase, and the obtained results (in terms of retention times and Rs, crit values) were compared to those obtained initially. The method repeatability was also checked by performing twenty consecutive injections from the same sample to demonstrate the robustness and stability of the retention times.

In the case of the Chirlacel OD column, setpoint 1 was selected because it could baseline separate six related substances (ezetimibe diol, desfluoro ezetimibe, THP compound, ezetimibe ketone, TBDMS ketone, and benzylated ezetimibe) and ezetimibe. The results (retention times and critical resolution) for the first and last injections are presented in Table 3; the mean values and standard deviations were calculated based on the 20 consecutive injections for the retention time of ezetimibe and for critical resolutions (R_s,crit.1_ resolution between desfluoro ezetimibe and ezetimibe API, R_s,crit.2_ resolution between ezetimibe API and THP compound).

In the case of the Chiral CD-Ph column, setpoint 3 was selected for applicability testing, and similarly as in the above case, the comparative results are presented in Table 4.

Based on the obtained results, the tested setpoints (setpoint 1 for Chiralcel OD column and setpoint 3 for Chiral CD-Ph column) show high robustness, without interference from the placebo components. The results indicate that these setpoints are relevant from a practical perspective and could be used for routine sample analysis. More specifically, the Chiralcel OD column with setpoint 1 conditions can be used for the determination of the following impurities: ezetimibe diol, desfluoro ezetimibe, THP compound, ezetimibe ketone TBDMS ketone, and benzylated ezetimibe. Chiral CD-Ph column with setpoint 3 conditions is suitable for the following impurities: ezetimibe diol, THP compound, RRS ezetimibe, ezetimibe ketone, TBDMS ezetimibe, and benzylated ezetimibe. The methods are robust, because the retention times and Rs value shifts were within acceptable ranges, even after repeating the analysis 2 years later.

## 3. Materials and Methods

### 3.1. Chemicals and Samples

#### 3.1.1. Chemicals

**Figure 5:**
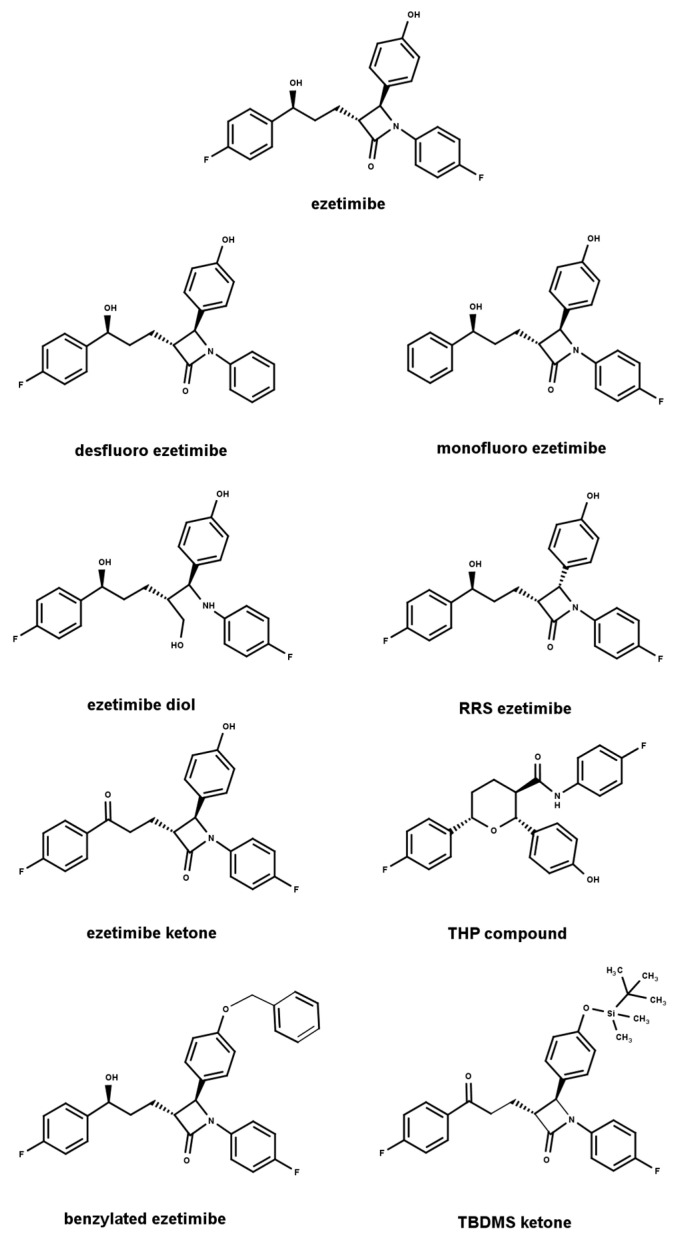
The chemical structure and the name used in the study for ezetimibe and its achiral-related substances.

Gradient-grade acetonitrile (ACN), methanol (MeOH), and glacial acetic acid (purity ≥ 99.7%) were used as mobile-phase components were purchased from Merck (Merck, Darmstadt, Germany). The purified water was freshly prepared each day by Millipore MilliQ Integral 10 (Millipore, Burlington, MA, USA) equipment. Ezetimibe was used as the active pharmaceutical ingredient (API) and its related substances (ezetimibe diol, monofluoro ezetimibe, desfluoro ezetimibe, RRS ezetimibe, ezetimibe ketone, THP (tetrahydropyran) compound, benzylated ezetimibe, and TBDMS (tert-butyldimethylsilyl) ketone) were obtained from LGC Standards (Teddington, London, UK). The chemical structure of the analytes represented by the Marvin Sketch software (version 17.27) and the chemical names used in this study are shown in Figure 5. The IUPAC names and the physical–chemical properties (logP and pKa) of the substances are summarized in Table S3.

#### 3.1.2. Artificial Samples for Retention Modeling

For retention modeling and computer-assisted peak tracking, three artificial sample solutions were used with the following compositions: artificial mixture 1 (mix1) containing all impurities and the API—ezetimibe 1000 µg/mL, ezetimibe diol 12 µg/mL, desfluoro ezetimibe 4 µg/mL, monofluoro ezetimibe 6 µg/mL, RRS ezetimibe 8 µg/mL, THP compound 6 µg/mL, ezetimibe ketone 8 µg/mL, benzylated ezetimibe 8 µg/mL, and TBDMS ketone 6 µg/mL. Artificial mixture 2 (mix2): ezetimibe diol 12 µg/mL, monofluoro ezetimibe 6 µg/mL, RRS isomer 8 µg/mL, ezetimibe ketone 8 µg/mL, benzylated ezetimibe 8 µg/mL, and TBDMS ketone 6 µg/mL. Artificial mixture 3 (mix3): desfluoro ezetimibe 4 µg/mL and THP compound 6 µg/mL. In all experimental runs for the construction of virtual retention models all three artificial mixture solutions were injected to ensure the proper tracking and identification of the closely- or co-eluted peaks (see Tables S1 and S2). ACN was used as a sample solvent for the preparation of solutions throughout the study and an injection volume of 10 μL was used.

### 3.2. Equipment and Software

The chromatographic measurements were carried out on a JASCO HPLC system (JASCO PU-2089 Plus quaternary gradient pump, AS-4050 autosampler, MD-2010 Plus diode array detector, and CO-2065 Plus column oven, Jasco Corporation, Tokyo, Japan). The software used to operate the equipment and data processing was ChromNAV (version 2.0, Jasco Corporation, Tokyo, Japan). The chromatograms were recorded at 247 nm, based on the UV spectrum of the ezetimibe.

The chemical structure of the molecules and the estimated physical–chemical parameters of the compounds (logP and pKa) were realized using MarvinSketch software (version 17.27, ChemAxon, Budapest, Hungary). The experimental design-based retention modeling, data analysis for method optimization, and in silico robustness testing were performed using the DryLab (version 4.5) modeling software package (Molnár-Institute, Berlin, Germany). The experimentally obtained chromatograms were exported into AIA/ANDI-format (*.cdf) and directly imported into the DryLab modeling software (version 4.5), providing the input data for peak tracking and for the construction of the virtual separation models.

### 3.3. Chromatographic Columns

Two different types of chiral selectors were selected as stationary phases. Firstly, as a reference column the Chiralcel OD 250 × 4.6 mm with 10 µm particles (Daicel Chiral Technologies Europe, Illkirck Cedex, France), based on the recommended L93 type column in ezetimibe API monograph of the USP [64]. This column is based on cellulose-tris(3,5-dimethylphenylcarbamate) chiral selector coated on silica gel particles. The secondary Chiral CD-Ph column has a packing material with phenylcarbamated-β-cyclodextrin chemically bonded to the surface of 5 µm spherical silica gel particles (Osaka Soda, Osaka, Japan). For better comparability, both columns shared the same dimensions (250 × 4.6 mm).

### 3.4. Preliminary Experiments and the Experimental Design Framework

Preliminary runs were realized to test the effect of the column temperature, gradient time, and organic composition of the mobile phase on chromatographic performance. As a reference, the Chiralcel OD column was used with neat MeOH and neat ACN as organic solvents, using different gradient times (from 10 to 80 min) and column temperatures (from 20 to 50 °C). The mix 1 solution was injected containing all the studied substances and the results were evaluated based on the number of separated peaks, resolution values, and the retention time of the last-eluting peak.

The experimental design framework was selected based on preliminary experiments and on our previous work on the separation of ezetimibe impurities using achiral stationary phases [9]. In the referenced study, the APPs were identified as the gradient program (including the time and the structure of the gradient, tG—gradient time), the ternary composition of the mobile phase (tC—ternary composition), and the column temperature (T—column temperature). It was also proved that the pH has no significant effect on the selectivity, probably because of the similar pKa value of the analytes [9]. Therefore, the three-dimensional *tG-T-tC* type design was chosen and realized on the selected CSPs. According to this experimental plan, the ternary composition was changed at three levels and the primary organic modifier was the ACN (B1); meanwhile, the secondary was the MeOH (B2) with lower eluotropic strength (tC_1_ = 100% B1, tC_2_ = 50% B2 in B1 and tC_3_ = 100% B2). The gradient time and the column temperature were changed systematically at two levels (tG_1_ = 20 min, tG_2_ = 60 min, T_1_ = 5 °C and T_2_ = 35 °C), needing 12 corner runs in total (one factor at three levels and two factors at two levels, 3^1^ × 2^2^ = 12) to build the virtual separation model. Ranges for the CMPs were selected based on preliminary runs, bearing in mind the recommendations of the DryLab software (version 4.5): a threefold difference between the gradient time levels tG_1_ and tG_2_ and a 30 °C difference between column temperatures T_1_ and T_2_. The structure of the experimental design is represented in Figure S2. For practical considerations, the flow rate used for the experimental runs was 0.5 mL/min to not exceed the pressure of 200 bars at low column temperatures.

## 4. Conclusions

Based on the obtained results, the mechanistic retention modeling approach can also be applied for the separation of achiral analytes with highly similar structures in reverse-phase mode.

The prediction accuracy of the virtual separation models is lower, mainly for the used polysaccharide CSP, but it can still be used to fine-tune method selectivity. This phenomenon is probably explicable through additional interactions or retention mechanisms apart from the reverse-phase mode. However, a much better correlation was observed between the predicted and experimentally verified retention times and resolution values for the cyclodextrin-based Chiral-CD-Ph column. The applied modeling approach ensures a high degree of flexibility, method understanding, and opportunities for control strategy, such as design space identification or column comparison, in accordance with the AQbD principles.

The construction of the virtual separation model is more complicated in the case of closely or co-eluted peaks, but the peak tracking process is achievable using the composite chromatogram function of the DryLab software (version 4.5). One of the biggest advantages of mechanistic modeling tools, such as the DryLab software (version 4.5), is the ability to visualize all possible cases through the covered knowledge space including virtual chromatograms, which is not feasible in the case of a purely statistics-based modeling approach.

Based on the results obtained during the applicability and robustness testing of the methods, it was demonstrated that the Chiralcel OD column with setpoint 1 and the Chiralcel CD-Ph column with setpoint 3 conditions can be orthogonally applied for the impurity profiling of ezetimibe. The methods proved to be robust not only on repetitive injections on the same day but also after long-term repetition, even 2 years after development.

## Figures and Tables

**Table 1:** Summary of the predicted and experimentally obtained data of the selected setpoints for the virtual separation model validation, in the case of the Chiralcel OD column.

Column		Chiralcel OD Column							
Setpoint No.		Setpoint 1		Setpoint 2		Setpoint 3		Setpoint 4	
**Conditions**		T = 15 °C Flow: 0.7 mL/min Gradient:		T = 20 °C Flow: 0.7 mL/min Gradient:		T = 30 °C Flow: 0.7 mL/min Gradient:		T = 20 °C Flow: 0.7 mL/min Gradient:	
		Time (min.)	Org. (%)	Time (min.)	Org. (%)	Time (min.)	Org. (%)	Time (min.)	Org. (%)
		0	26	0	26	0	34	0	50
		70	86	40	86	24	53	50	100
				45	86	40	95	60	100
						45	95		
		tC: 100% ACN		tC: 100% ACN		tC: 100% ACN		tC: 100% MeOH	
**R_t_ (min.)**		Drylab	Exp.	Drylab	Exp.	Drylab	Exp.	Drylab	Exp.
**Compounds**	**ezetimibe diol**	23.72	25.73	20.19	20.12	17.50	17.57	27.29	25.00
	**desfluoro ezetimibe**	32.20	34.86	25.72	25.48	-	-	-	-
	**ezetimibe API**	33.97	36.16	26.70	26.25	25.70	27.81	36.61	35.91
	**THP compound**	35.79	38.17	27.80	27.33	27.55	30.06	-	-
	**monofluoro ezetimibe**	-	-	-	-	-	-	38.28	37.26
	**ezetimibe ketone**	41.79	44.59	31.80	31.40	32.27	35.40	45.92	46.41
	**TBDMS ketone**	56.38	58.33	40.10	39.57	39.38	41.58	50.29	52.44
	**benzylated ezetimibe**	63.41	65.94	44.31	43.62	42.26	43.60	52.23	55.50
**R_s,crit._**		1.60	1.63	1.23	1.29	2.35	2.56	1.52	1.56
**Average of retention time errors (%)**		5.88		1.27		5.59		4.15	

**Drylab**—retention time predicted by the DryLab software; **Exp.**—experimentally obtained retention time; **Retention time error (%)** = (DryLab R_t_ − Exp. R_t_)/Exp. R_t_ × 100; **R_t_**—retention time; **R_s,crit._**—critical resolution between the closest peaks; **T**—column temperature; **tC**—ternary composition.

**Table 2:** Summary of the predicted and experimentally obtained data of the selected setpoints for model validation in the case of the Chiral CD-Ph column.

Column		Chiral CD-Ph Column							
Setpoint No.		Setpoint 1		Setpoint 2		Setpoint 3		Setpoint 4	
**Conditions**		T = 5 °C Flow: 0.5 mL/min Gradient:		T = 5 °C Flow: 0.5 mL/min Gradient:		T = 5 °C Flow: 0.7 mL/min Gradient:		T = 5 °C Flow: 0.5 mL/min Gradient:	
		Time (min.)	Org. (%)	Time (min.)	Org. (%)	Time (min.)	Org. (%)	Time (min.)	Org. (%)
		0	60	0	50	0	50	0	50
		50	100	30	100	30	100	40	100
		55	100	42	100	40	100		
		tC: 100% MeOH		tC: 100% MeOH		tC: 100% MeOH		tC: 50% MeOH in ACN	
**R_t_ (min.)**		Drylab	Exp.	Drylab	Exp.	Drylab	Exp.	Drylab	Exp.
**Compounds**	**ezetimibe diol**	22.22	20.48	23.02	23.11	19.05	18.32	18.97	18.06
	**THP compound**	27.76	26.46	26.19	26.59	22.37	21.89	-	-
	**RRS ezetimibe**	31.02	30.10	28.07	28.51	24.17	23.79	-	-
	**monofluoro ezetimibe**	32.38	31.22	-	-	-	-	-	-
	**ezetimibe API**	33.85	33.16	29.79	30.00	25.74	25.20	23.96	23.60
	**ezetimibe ketone**	41.13	40.78	34.06	34.19	29.70	29.63	28.55	28.35
	**TBDMS ketone**	49.30	50.45	38.73	37.85	33.93	34.68	37.13	37.62
	**benzylated ezetimibe**	50.83	52.08	41.10	39.11	35.15	35.34	34.76	35.07
**R_s,crit._**		1.36 * 1.42 **	1.33 * 1.44 **	1.99	1.75	2.09	1.80	-	-
**Average of retention time errors (%)**		3.47		1.70		1.84		1.89	

**Drylab**—retention time predicted by the DryLab software; **Exp.**—experimentally obtained retention time; **Retention time error (%)** = (DryLab R_t_ − Exp. R_t_)/Exp. R_t_ × 100; **R_t_**—retention time; **R_s,crit._**—critical resolution between the closest peaks; **T**—column temperature; **tC**—ternary composition. * R_s,crit.__1_ critical resolution between RRS ezetimibe and monofluoro ezetimibe. ** R_s,crit.2_ critical resolution between monofluoro ezetimibe and ezetimibe API.

**Table 3:** The applicability of setpoint 1 in the case of Chiralcel OD column.

Column		Chiralcel OD Column			
Setpoint No.		Setpoint 1		Setpoint 1	Setpoint 1
**Measurement**		After development		2 years after development	2 years after development
**Sample**		Artificial mixture without placebo		1st injection of artificial sample containing placebo components	20th injection of artificial sample containing placebo components
**R_t_ (min.)**		Drylab	Exp.	Exp.	Exp.
**Compounds**	**ezetimibe diol**	23.72	25.73	25.57	25.65
	**desfluoro ezetimibe**	32.20	34.86	34.78	34.87
	**ezetimibe API**	33.97	36.16	35.99	36.06
	**THP compound**	35.79	38.17	37.91	37.98
	**ezetimibe ketone**	41.79	44.59	44.55	44.61
	**TBDMS ketone**	56.38	58.33	58.30	58.36
	**benzylated ezetimibe**	63.41	65.94	65.75	65.81
**R_s,crit.1_**		1.60	1.63	2.02	1.99
**R_s,crit.2_**		1.69	2.26	2.77	2.77
**Average of retention time errors (%)**		5.88		5.55	5.73
**Mean ± SD (20 injections)**		Rt_ezetimibe_		36.02 ± 0.20	
		R_s,crit.1_		1.99 ± 0.02	
		R_s,crit.2_		2.71 ± 0.07	

**R_s,crit.1_**—resolution between desfluoro ezetimibe and ezetimibe API; **R_s,crit.2_**—resolution between ezetimibe API and THP compound; **SD**—standard deviation; **Drylab**—retention time predicted by the DryLab software; **Exp.**—experimentally obtained retention time; **Retention time error (%)** = (DryLab Rt − Exp. Rt)/Exp. Rt × 100; **Rt**—retention time.

**Table 4:** The applicability of setpoint 3 in the case of Chiral CD-Ph column.

Column		Chiral CD-Ph Column			
Setpoint no.		Setpoint 3		Setpoint 3	Setpoint 3
**Measurement**		After Development		2 years after development	2 years after development
**Sample**		Artificial mixture without placebo		1st injection of artificial sample containing placebo components	20th injection of artificial sample containing placebo components
**R_t_ (min.)**		Drylab	Exp.	Exp.	Exp.
**Compounds**	**ezetimibe diol**	19.05	18.32	19.27	19.31
	**THP compound**	22.37	21.89	22.72	22.81
	**RRS ezetimibe**	24.17	23.79	24.52	24.61
	**ezetimibe API**	25.74	25.20	25.99	26.09
	**ezetimibe ketone**	29.70	29.63	29.88	29.95
	**TBDMS ketone**	33.93	34.68	34.27	34.36
	**benzylated ezetimibe**	35.15	35.34	35.38	35.48
**R_s,crit._**		2.09	1.80	1.62	1.63
**Average of retention time errors (%)**		1.84		1.04	1.35
**Mean ± SD (20 injection)**		Rt_ezetimibe_		26.03 ± 0.16	
		R_s,crit._		1.63 ± 0.06	

**R_s,crit._**—resolution between RRS ezetimibe and ezetimibe API; **SD**—standard deviation; **Drylab**—retention time predicted by the DryLab software; **Exp.**—experimentally obtained retention time; **Retention time error (%)** = (DryLab Rt − Exp. Rt)/Exp. Rt × 100; **Rt**—retention time.

## Data Availability

The data presented in this study are available on request from the corresponding author.

## Supplementary Materials

The following supporting information can be downloaded at: https://www.mdpi.com/article/10.3390/ijms242216097/s1.

## Abbreviations

ACN—acetonitrile; API—active pharmaceutical ingredient; APP—analytical procedure parameter; AQbD—analytical quality by design; ATP—analytical target profile; CD—cyclodextrin; CMP—critical method parameter; CQA—critical quality attribute; CSP—chiral stationary phase; DoE—design of experiments; DS—design space; DSC—design space comparison; EC—established condition; Exp.— experimental; HIC—hydrophobic interaction chromatography; HILIC—hydrophobic interaction liquid chromatography; HPLC—high-performance liquid chromatography; ICH—International Council for Harmonization; ICH Q2(R2)—ICH guideline on validation of analytical procedures (under revision); ICH Q14—ICH guideline Q14 on analytical method development (under revision); IEX—ion exchange chromatography; IUPAC—International Union of Pure and Applied Chemistry; LSSM—linear solvent strength model; MeOH—methanol; MODR—method operational design region; PAR—proven acceptable range; QbD—quality by design; R_s,crit._—critical resolution; Rt—retention time; T—column temperature; TBDMS—tert-butyldimethylsilyl; tC-ternary composition; tG—gradient time; THP—tetrahydropyran; USP—United States Pharmacopeia.
