# Supplementary material for: The Applicability of Chromatographic Retention Modeling on Chiral Stationary Phases in Reverse-Phase Mode: A Case Study for Ezetimibe and Its Impurities
Source: Int J Mol Sci. 2023 Nov 8;24(22):16097. doi: 10.3390/ijms242216097 (PMC10671152; doi:10.3390/ijms242216097)
Supplement: Supplementary file 1 [file ijms-24-16097-s001.zip › ijms-2641814-supplementary.docx]

Article

The Applicability of Chromatographic Retention Modeling on Chiral Stationary Phases in Reverse-Phase Mode: A Case Study for Ezetimibe and Its Impurities

**Table S1**. The obtained retention times in the 12 corner conditions of *tG-T-tC* experimental design for all examined substances in the case of the Chiralcel OD column, arranged generally by the elution order.

| CMPs | Ternary composition  (%) | 100 % B1  (ACN) | | | | 50 % B2 in B1  (50 % MeOH in ACN) | | | | 100 % B2  (MeOH) | | | |
| --- | --- | --- | --- | --- | --- | --- | --- | --- | --- | --- | --- | --- | --- |
|  | **Gradient time**  **(min.)** | **20** | **60** | **20** | **60** | **20** | **60** | **20** | **60** | **20** | **60** | **20** | **60** |
|  | **Column temperature**  **(°C)** | **5** | | **35** | | **5** | | **35** | | **5** | | **35** | |
|  | **No. of corner run** | **1** | **2** | **3** | **4** | **5** | **6** | **7** | **8** | **9** | **10** | **11** | **12** |
| **Substances** | ezetimibe diol | 16.70 | 24.36 | 16.29 | 23.05 | 21.06 | 36,03 | 20.02 | 32.57 | 25.11 | 48.10 | 23.64 | 43.06 |
|  | desfluoro ezetimib | 19.15 | 30.98 | 18.68 | 29.53 | 23.85 | 44.33 | 22.53 | 40.81 | 27.67 | 56.68 | 26.25 | 52.38 |
|  | RRS ezetimibe | 19.15 | 31.38 | 18.81 | 30.01 | 23.72 | 45.08 | 22.71 | 41.60 | 27.72 | 57.31 | 26.44 | 53.01 |
|  | **ezetimibe (API)** | **19.45** | **32.19** | **18.95** | **30.55** | **23.90** | **45.30** | **22.80** | **41.55** | **27.74** | **57.00** | **26.40** | **52.55** |
|  | THP compound | 19.72 | 33.23 | 19.37 | 32.08 | 23.85 | 45.62 | 22.81 | 42.10 | 27.21 | 56.01 | 26.01 | 51.92 |
|  | monofluoro ezetimib | 20.26 | 33.33 | 19.40 | 31.08 | 24.44 | 46.67 | 23.15 | 42.49 | 28.44 | 58.84 | 26.96 | 53.94 |
|  | ezetimibe ketone | 22.26 | 38.86 | 21.38 | 36.88 | 26.49 | 53.22 | 25.18 | 48.83 | 33.28 | 67.45 | 30.02 | 61.65 |
|  | benzylated ezetimibe | 26.15 | 49.46 | 25.02 | 47.21 | 28.80 | 60.80 | 27.65 | 57.46 | 36.37 | 71.42 | 32.56 | 67.15 |
|  | TBDMS ketone | 27.41 | 54.64 | 26.41 | 52.39 | 29.66 | 63.61 | 28.49 | 60.82 | 38.01 | 73.65 | 33.76 | 69.35 |

**Table S2**. The obtained retention times in the 12 corner conditions of *tG-T-tC* experimental design for all examined substances in the case of the Chiral CD-Ph column, arranged generally by the elution order.

| CMPs | Ternary composition  (%) | 100 % B1  (ACN) | | | | 50 % B2 in B1  (50 % MeOH in ACN) | | | | 100 % B2  (MeOH) | | | |  |
| --- | --- | --- | --- | --- | --- | --- | --- | --- | --- | --- | --- | --- | --- | --- |
|  | **Gradient time**  **(min.)** | **20** | **60** | **20** | **60** | **20** | **60** | **20** | **60** | **20** | **60** | **20** | **60** |  |
|  | **Column temperature**  **(°C)** | **5** | | **35** | | **5** | | **35** | | **5** | | **35** | |  |
|  | **No. of corner run** | **1** | **2** | **3** | **4** | **5** | **6** | **7** | **8** | **9** | **10** | **11** | **12** |  |
| **Substances** | ezetimibe diol | 16.76 | 26.50 | 16.48 | 24.65 | 20.35 | 34.55 | 19.17 | 31.10 | 23.34 | 44.00 | 22.08 | 38.44 | |
|  | desfluoro ezetimib | 18.50 | 31.20 | 18.09 | 30.04 | 22.27 | 41.15 | 21.15 | 37.56 | 26.61 | 54.22 | 25.04 | 47.76 | |
|  | monofluoro ezetimib | 18.46 | 31.61 | 18.01 | 29.78 | 22.34 | 41.04 | 21.02 | 37.53 | 26.65 | 52.99 | 24.72 | 46.94 | |
|  | RRS ezetimibe | 18.53 | 31.83 | 18.01 | 30.10 | 22.34 | 41.07 | 21.02 | 37.53 | 25.67 | 52.08 | 24.39 | 46.54 | |
|  | **ezetimibe API** | **18.46** | **31.90** | **18.18** | **30.15** | **22.34** | **41.55** | **21.15** | **37.86** | **26.65** | **54.30** | **25.04** | **47.88** | |
|  | THP compound | 18.89 | 33.60 | 18.60 | 31.83 | 22.27 | 41.54 | 21.10 | 38.00 | 24.69 | 49.45 | 23.75 | 44.67 | |
|  | ezetimibe ketone | 20.07 | 36.31 | 19.64 | 34.42 | 24.21 | 46.93 | 22.86 | 42.79 | 29.01 | 59.98 | 27.08 | 53.47 | |
|  | benzylated ezetimibe | 22.50 | 43.14 | 22.06 | 41.61 | 26.30 | 54.14 | 24.95 | 50.38 | 32.66 | 67.51 | 28.97 | 61.81 | |
|  | TBDMS ketone | 23.69 | 47.23 | 23.19 | 45.59 | 26.94 | 56.86 | 25.67 | 53.21 | 31.16 | 66.14 | 29.75 | 61.28 | |

**Table S3**. The IUPAC names, chemical formulas, molecular weights and the calculated physical-chemical properties (logP and pKa) of the ezetimibe and its related substances used in the study. The logP and pKa values were calculated by the Marvin Sketch software.

| Name used in the study, chemical formula and molecular weight | IUPAC name | logP | pKa  (phenolic hydroxyl) |
| --- | --- | --- | --- |
| **ezetimibe**  C_24_H_21_F_2_NO_3_  M=409.43 | (3R,4S)-1-(4-fluorophenyl)-3-[(3S)-3-(4-fluorophenyl)-3-hydroxypropyl]-4-  (4-hydroxyphenyl)azetidin-2-one | 4.63 | 9.49 |
| **ezetimibe diol**  C_24_H_25_F_2_NO_3_  M=413.47 | (3R,4S)-1-(4-fluorophenyl)-(4-  hydroxymethyl)-5-(4-hydroxyphenyl)-5-N-  (4-fluorophenylamino)-pentanol | 4.47 | 9.49 |
| **desfluoro ezetimibe**  C_24_H_22_FNO_3_  M=391.44 | (3R,4S)-3-[(3S)-3-(4-fluorophenyl)-3-hydroxypropyl]-4-(4-hydroxyphenyl)-1-phenyl-2-azetidinone | 4.49 | 9.48 |
| **RRS ezetimibe**  C_24_H_21_F_2_NO_3_  M=409.43 | (3R,4R)-1-(4-fluorophenyl)-3-[(3S)-3-(4-fluorophenyl)-3-hydroxypropyl]-4-  (4-hydroxyphenyl)azetidin-2-one | 4.49 | 9.48 |
| **monofluoro ezetimibe**  C_24_H_22_FNO_3_  M=391.44 | (3R,4S)-1-(4-fluorophenyl)-3-((S)-3-hydroxy-3-phenylpropyl)-4-(4-hydroxyphenyl)azetidin-2-one | 4.49 | 9.48 |
| **ezetimibe ketone**  C_24_H_19_F_2_NO_3_  M=407.42 | (3R,4S)-1-(4-fluorophenyl)-3-[3-(4-fluorophenyl)-3-oxopropyl]-4-  (4-hydroxyphenyl)azetidin-2-one | 4.52 | 9.48 |
| **THP (tetrahydropyran) compound**  C_24_H_21_F_2_NO_3_  M=409.43 | (2R,3R,6S)-N,6-bis(4-fluorophenyl)-2-(4-hydroxyphenyl)oxane-3-carboxamide | 5.44 | 9.47 |
| **benzilated ezetimibe**  C_31_H_27_F_2_NO_3_  M=499.57 | (3R,4S)-1-(4-fluorophenyl)-3-[(3S)-3-(4-  fluorophenyl)-3-hydroxyphenyl]-4-(4-  benzyloxyphenyl)-2-azetidinone | 6.44 | - |
| **TBDMS (tert-butyldimethylsilyl) ketone**  C_30_H_33_F_2_NO_3_Si  M=521.68 | (3R,4S)-1-(4-fluorophenyl)-3-[(3S)-3-(4-fluorophenyl)-3-hydroxypropyl]-4-  (4- tert-butyldimethylsilyl-phenyl)azetidin-2-one | 6.74 | - |


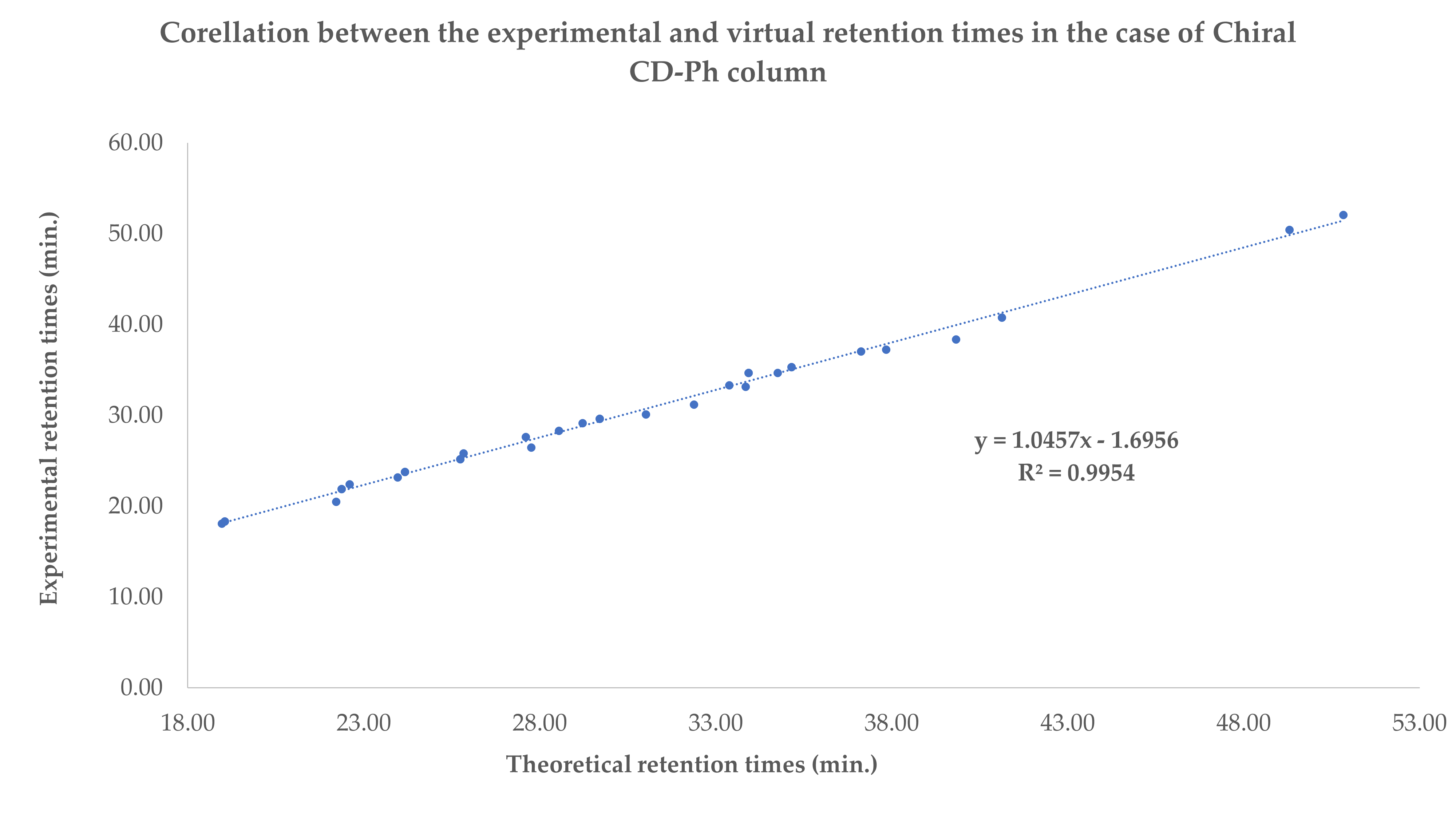


**Figure S1**. Correlation between the experimental and virtual retention times in the case of the Chiral CD-Ph column for all four tested setpoints


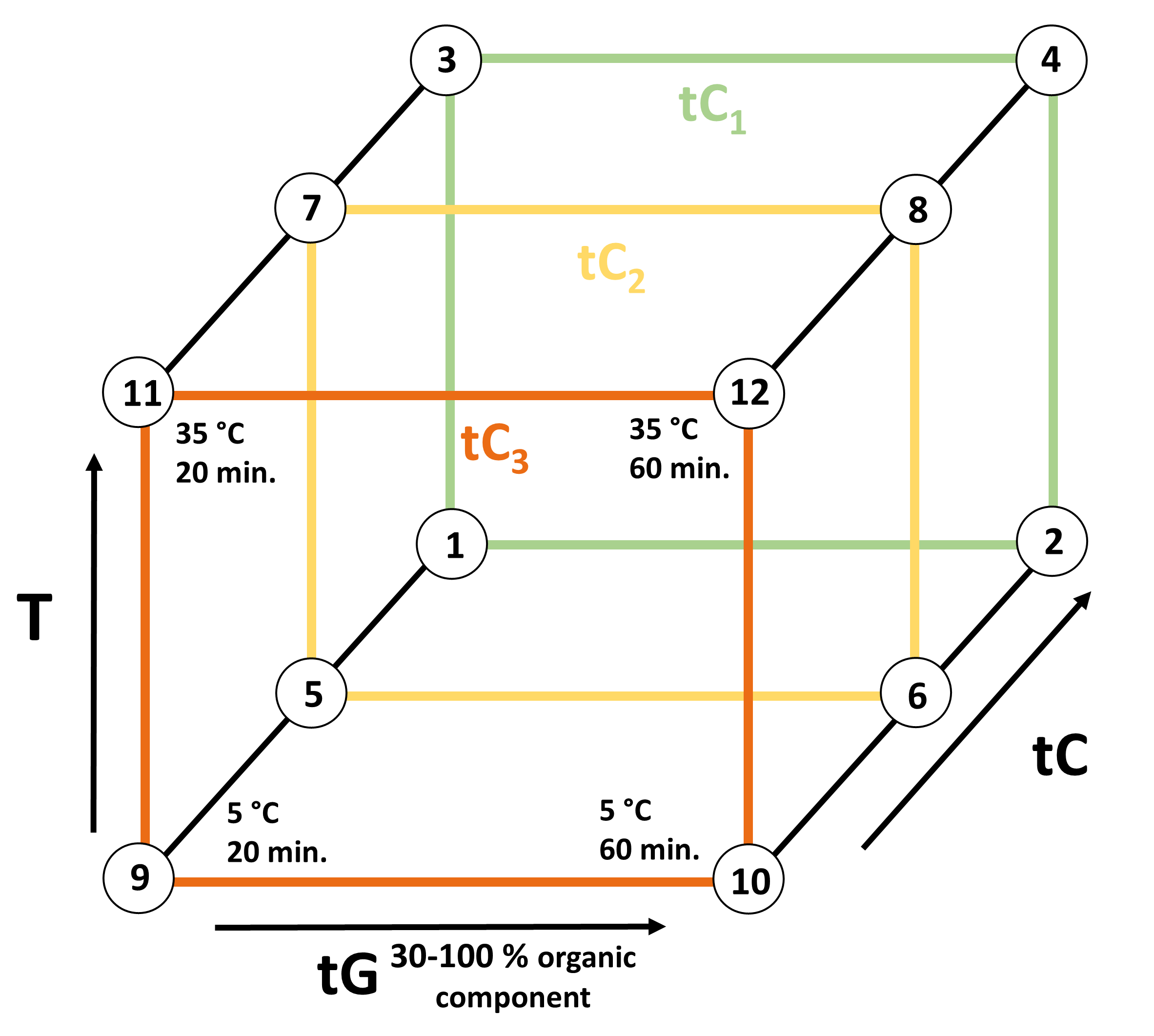


**Figure S2**. The structure of the three-dimensional *tG-T-tC* experimental design including the levels and the ranges of the modelled method parameters. The number of corner runs represents the recommended order by the DryLab software for experimental runs.
